# Common mental disorders and associated factors in nursing workers in COVID-19 units

**DOI:** 10.1590/1980-220X-REEUSP-2022-0059en

**Published:** 2022-08-05

**Authors:** Alexa Pupiara Flores Coelho Centenaro, Andressa de Andrade, Gianfábio Pimentel Franco, Leticia Silveira Cardoso, Lílian Moura de Lima Spagnolo, Rosângela Marion da Silva

**Affiliations:** 1 Universidade Federal de Santa Maria, Departamento de Ciências da Saúde, Palmeira das Missões, RS, Brazil.; 2 Universidade Federal do Pampa, Departamento de Enfermagem, Uruguaiana, RS, Brazil.; 3 Universidade Federal de Pelotas, Departamento de Enfermagem, Pelotas, RS, Brazil.; 4 Universidade Federal de Santa Maria, Departamento de Enfermagem, Santa Maria, RS, Brazil.

**Keywords:** Nursing, Ocuppational Health, COVID-19, Pandemics, Nursing Practitioners, Hospital Units, Enfermería, Salud Laboral, COVID-19, Pandemias, Enfermeras Practicantes, Unidades Hospitalarias, Enfermagem, Saúde do Trabalhador, COVID-19, Pandemias, Profissionais de Enfermagem, Unidades Hospitalares

## Abstract

**Objective:**

To analyze the interfaces between mental illness, based on common mental disorder screening, and sociodemographic, health and life habits aspects of nursing workers at COVID-19 units.

**Method:**

A mixed methods study, carried out with 327 nursing workers from COVID-19 units of seven public and philanthropic, medium and large hospitals in Brazil. The collection included a socio-employment, health and lifestyle questionnaire, the Self-Reporting Questionnaire, and interviews. chi-square and Fisher’s exact tests were applied to quantitative data and thematic content analysis, with the help of NVivo in the qualitative ones.

**Results:**

Common mental disorders were screened in 35.5% of the sample and were associated with female sex (p = 0.004), age up to 40 years (p = 0.003), nurse (p = 0.014), reporting previous illness (p = 0.003), using psychoactive drugs (p < 0.001), medication that was not used before the pandemic (p < 0.001) and reporting poor sleep/eating quality (p < 0.001). The impacts of the pandemic on social and family life presented interfaces with mental illness.

**Conclusion:**

The presence of psychological illness is suggested, possibly associated with the repercussions of the pandemic on work and personal life.

## RESUMO

**Objetivo:**

Analisar as interfaces entre o adoecimento mental, a partir do rastreamento de transtornos mentais comuns, e aspectos sociodemográficos, de saúde e hábitos de vida de trabalhadores de enfermagem de unidades COVID-19.

**Método:**

Estudo de métodos mistos, realizado com 327 trabalhadores de enfermagem de unidades COVID-19 de sete hospitais do Brasil, públicos e filantrópicos, de médio e grande porte. A coleta incluiu um questionário sociolaboral, de saúde e hábitos de vida, o *Self-Reporting Questionnaire*, e entrevistas. Foram aplicados Testes Qui-Quadrado e Exato de Fisher nos dados quantitativos e análise temática de conteúdo, com auxílio do *software* NVivo nos qualitativos.

**Resultados:**

Os transtornos mentais comuns foram rastreados em 35,5% da amostra e se associaram ao sexo feminino (p = 0,004), idade de até 40 anos (p = 0,003), enfermeiro (p = 0,014), relatar doença prévia (p = 0,003), usar psicoativos (p < 0,001), medicamentos que não utilizava antes da pandemia (p < 0,001) e relatar má qualidade do sono/alimentação (p < 0,001). Os impactos da pandemia na vida social e familiar apresentaram interfaces com o adoecimento mental.

**Conclusão:**

Sugere-se a presença de adoecimento psíquico, possivelmente associado às repercussões da pandemia no trabalho e na vida pessoal.

## RESUMEN

**Objetivo:**

Analizar las interfaces entre la enfermedad mental, a partir del tamizaje de los trastornos mentales comunes, y aspectos sociodemográficos, de salud y de hábitos de vida de trabajadores de enfermería en unidades de COVID-19.

**Método:**

Estudio de métodos mixtos, realizado con 327 trabajadores de enfermería de unidades COVID-19 de siete hospitales públicos y filantrópicos, medianos y grandes de Brasil. La recogida incluyó un cuestionario sociolaboral, de salud y de estilo de vida, el Self-Reporting Questionnaire y entrevistas. Para los datos cuantitativos y análisis de contenido temático se aplicaron las pruebas Chi-Cuadrado y Exacto de Fisher, con la ayuda del software NVivo en los cualitativos.

**Resultados:**

Los trastornos mentales comunes fueron tamizados en el 35,5% de la muestra y se asociaron al sexo femenino (p = 0,004), edad hasta 40 años (p = 0,003), enfermero (p = 0,014), relatar enfermedad previa (p = 0,003), usar psicofármacos (p < 0,001), medicación que no se usaba antes de la pandemia (p < 0,001) y referir mala calidad del sueño/alimentación (p < 0,001). Los impactos de la pandemia en la vida social y familiar presentaron interfaces con la enfermedad mental.

**Conclusión:**

Se sugiere la presencia de enfermedad psíquica, posiblemente asociada a las repercusiones de la pandemia en la vida laboral y personal.

## INTRODUCTION

Work is considered a human action that involves the engagement of body, mind and psychological and affective abilities. In addition to an action with a productive purpose, it is considered an experience that produces health or physical, mental and social illness. In this perspective, this study is based on the theoretical-conceptual conception that considers workers’ health from the interface between work, subjectivity and health^(1)^.

With regard to mental illness at work, common mental disorders (CMD), which are characterized by a group of recognized non-psychotic symptoms, such as depressed mood, anxiety, insomnia, fatigue, irritability, memory and concentration deficits, which result in mental illness^(2,3)^. This set of symptoms has been associated with health care workers^(4,5)^.

It is estimated that the prevalence of CMD in the world is around 24.6% to 45.3%, and in Brazil, between 28.7 and 50%^(6,7)^. Recent studies, which investigated the prevalence of CMD in nursing workers in a hospital context, indicated an oscillation between 26% and 58%^(3–5,8)^, an aspect that generates an alert in the sense of health surveillance.

Nursing currently represents the largest workforce in the field of health, at a global level^(9)^. The work process in this environment has peculiar characteristics and has often been linked to mental illness, the latter being associated with the complexity of professional performance, excessive working hours, precarious working conditions and low pay^(5,10)^.

In 2019, the emergence of the pandemic caused by the new coronavirus (COVID-19) and the triggering of a health alert worldwide put the role of nursing in vogue, especially for professionals who work on the front line in assisting users. This scenario has been pointed out as a potentiator of the impacts on workers’ mental health, evidencing an important illness in this context^(11,12)^.

Studies have highlighted a higher level of professional stress caused by COVID-19 related to the accelerated pace of spread of the disease and the restructuring of services. The high workload, daily coping with situations of illness and death, the possibility of self-contamination, social distancing and concern for family members are also mentioned. In these studies, symptoms such as anxiety, anguish, depression, fear and sleep disturbances were highlighted^(12,13,14)^.

Organizational aspects are also identified as generators of stress among workers. Human resource reallocation, concern with lack of personal protective equipment (PPE), lack of knowledge about the disease, rapid changes in the direction of care conducts, absence of specific medications and shortage of respirators and beds, especially intensive care ones^(11)^.

Thus, it is believed that aspects of nursing work that favored workers’ mental illness have presented an exacerbation during the pandemic period. This aspect, combined with the scarcity of publications that focus on CMDs and the interface with nursing workers who work in COVID-19 units in Brazil, points to the need for research that evidence this condition, seeking to mobilize society to promote actions that contribute to the minimization of illness factors and benefit workers.

Considering the above, this study aimed to analyze the interfaces between mental illness, based on CMD screening, and sociodemographic, health and lifestyle aspects of nursing workers at COVID-19 units.

## METHOD

### Type of Study

This is a multicenter, mixed-methods study, with concomitant triangulation of quantitative and qualitative data. The research process involved a quantitative cross-correlational stage and a qualitative descriptive stage.

### Setting, Participants and Selection Criteria

The scenarios were seven hospital institutions from different regions of Rio Grande do Sul, Brazil. Four were large and three were medium. Five were philanthropic institutions. Two were characterized as university hospitals, linked to federal educational institutions and exclusively serving the Unified Health System (*Sistema Único de Saúde*). The seven institutions were references in their regions for the care of serious cases of COVID-19. The units that were part of the flow of care for COVID-19 patients were included, totaling one respiratory triage unit, five urgency and emergency sectors, four clinical inpatient units and four Intensive Care Units (ICU).

Participants were nursing workers (nurses and technicians, categories present in these scenarios during the study period), assigned to these units (eligibility criteria). Professionals on vacation or on leave during the data collection period were excluded. The eligible population consisted of 470 workers.

The adequate sample was estimated from the sample calculation for prevalence research. In this regard, considering a population of 470 workers, with an estimated frequency of 50%, a confidence level of 95%, a margin of error of 5%, effect design and cluster 1, a minimum number of 211 participants was estimated. All workers who met the eligibility criteria were invited.

### Data Collection

The research took place between September 2020 and July 2021. The quantitative stage included a self-administered instrument, available on the free digital platform Google Forms (G Suite^®^ tool). First, there was the Informed Consent Form (ICF), followed by the option to sign the consent, which allowed access to the questions. The questionnaire included variables for surveying socio-employment data prepared by the research team as: sex; age; marital status; race/color; weekly workload; employment relationship; position/role; unit in which it was assigned; work shift; and job tenure. Moreover, it included health variables and life habits as: presence of diseases; presence of more than one type of associated disease; smoking; practice of physical exercises; use of medications that were not used before the pandemic; use of analgesics, antipyretics and anti- inflammatory drugs; use of psychoactives; use of more than one class of medication, including psychoactives; self-assessment of satisfaction with sleep and diet; whether they were part of a risk group for COVID-19; and if they had already tested positive for COVID-19. All data were based on participants’ self-report.

The questionnaire also contained the Self-Reporting Questionnaire (SRQ-20), a validated instrument used to assess mental health through CMD screening and composed of 20 questions, aimed at non-psychotic symptoms, which include insomnia, fatigue, irritability, forgetfulness, difficulty concentrating, and somatic complaints. Responses were dichotomized into “yes” and “no”. The results may suggest a suspicion of CMD, through the identification of symptoms that do not result in a diagnosis^(15)^.

Quantitative collection followed a protocol for sending the questionnaire to these contacts, with the intermediary of nursing managers who contributed to research dissemination and team mobilization. One of the institutions requested that data collection was in person. In this case, the questionnaires were delivered to participants at their workplace, packed in an envelope with the informed consent in two copies. Collection was made at the workplace by appointment with each worker.

The qualitative stage included individual semi-structured interviews with a sample of five nursing workers from each hospital institution (35 in total), selected through a simple random draw. The first interview was considered a pilot for adjustments to the semi-structured script. As no changes to the script were necessary, it was added to the database. The material obtained in the 35 interviews reached the theoretical saturation criteria. This step was conducted by a team of professors and master’s students in nursing, all with experience in field research.

In five hospital institutions, interviews were carried out face-to-face, during working hours, by means of an agenda with workers. They were conducted by the researchers in airy, safe and private environments, with all necessary precautionary measures. The semi-structured script included questions related to participants’ perception of their work with COVID-19 units and interfaces with their mental health.

Two institutions requested that the interviews be conducted online. For this, the Google Meet digital platform (G Suite^®^ tool) was used. No participant had difficulty accessing and using the platform. The online interview script was identical to the face-to-face script.

The interviews lasted a mean of 22.5 minutes. They were recorded with the consent of all participants and transcribed in full. The transcripts made up the qualitative corpus of the study.

### Data Analysis

Quantitative analysis was performed by tabulating and coding the data in an Excel spreadsheet and later transferring it to SPSS, version 20.0. Missing data, as well as variables with data below the minimum sample population or greater than 10%, were not considered in the analysis. Means, absolute (n) and relative (%) frequencies were used to describe the sample. For CMD screening, the number of individual affirmative responses in the SRQ-20 was counted. The score ranges from 0, indicating low probability of CMD, to 20 indicating high probability of CMD. The cut-off point used was 6 affirmative responses for males and 8 for females^(16)^. Positive results for CMD screening (outcome variable) were associated with socio-occupational, health and lifestyle variables (exposure variables), using Pearson’s chi-square test and Fisher’s exact test. Throughout the entire analysis, a statistical significance level of 5% was adopted.

Qualitative data were submitted to thematic content analysis^(17)^, which is carried out in three stages. The first stage, pre-analysis, started with text skimming to select material relevant to the study’s objective. The second stage, material exploration, included testimony decomposition and coding in recording units, with the help of NVivo.

The last stage, data processing and interpretation, included theorizing from triangulation with the results of the quantitative stage. These results were approximated and compared, establishing relationships of complementarity that made it possible to reach the study’s objective.

### Ethical Aspects

In the presentation of results, the deponents were identified by the letter W (for “worker”), followed by a random cardinal number. Throughout the study, the ethical precepts established by Resolutions 466/2012 and 510/2016 of the Brazilian National Health Council were observed. The project was approved by a local Research Ethics Committee in 2020, with Protocol 4,206,065, with amendments approved in 2020 (Procols 4,363,162 and 4,395,923) and 2021 (Protocol 4,549,077).

## RESULTS

Of an eligible population of 470 workers, there were 111 refusals (the main reasons reported were the overload of research aimed at this population in the period) and 32 losses attributed to incomplete questionnaires. Therefore, 327 nursing workers participated in the quantitative stage, totaling about 70% of eligible population.

In the quantitative stage, female individuals (n = 278, 85%) prevailed, with a mean age of 35.3 (±9.3) years, white (n = 260, 79.5%), married or in a stable union (n = 175, 53.5%). There was a predominance of nursing technicians (n = 250, 76.4%), linked to the Consolidation of Labor Laws (CLT) regime (n = 212, 64.8%), with 5 years or more of experience (n = 213, 65.1%) and a workload of up to 40 hours per week (n = 200, 61.1%). In the qualitative stage, among the 35 workers, there was a predominance of women (n = 28, 80%), white (n = 27, 77%), nursing technicians (n = 25, 71.4%) and with a mean of 38 (±8.9 years).

The factors associated with CMD are described in the following two axes: *Common mental disorders and interface with socio-occupational characteristics of nursing workers in COVID-19 units*; and *Common mental disorders and interface with nursing workers’ health conditions and life habits in COVID-19 units*.

### Common Mental Disorders and Interface with Socio-Occupational Characteristics of Nursing Workers in Covid-19 Units

The prevalence of workers screened for CMD, as a result of SRQ-20, was 35.5% (n = 116). The interviews contributed to the understanding that working on the front line was related to psychological damage in part of participants and, in some cases, a desire to leave the profession:

(…) *I have experienced many things here that I never imagined* (…) *a whirlwind of emotions* (…) *my anxiety got a lot worse* (…) *because I like to have results and here* [COVID-19 unit] *everything is very slow… so the impact on my mental health was very huge* (…) (W4).

(…) *It’s been very difficult psychologically. We see so many people, even young people dying. It’s difficult.* (…) *it’s very stressful, I’m very tired. I don’t know if I will continue in the profession* (…) (W14).

Variable CMD presented statistical significance, when associated with the female sex, to workers aged up to 40 years and who were nurses, as evidenced in the Table 1 below.

**Table 1.** Common mental disorders and the association with socio-occupational characteristics of nursing workers in COVID-19 units – Rio Grande do Sul, Brazil, 2021 (n = 327)*.

Variable	CMD screening^†^ (n = 327)				p-value
	No (n = 211)		Yes (n = 116)		
	N	%	N	%
Sex					**0.004** ^‡^
Female	171	81.0	107	92.2	
Male	40	19.0	9	7.8	
Age group (n=287)					**0.003** ^§^
Up to 40 years	117	63.9	83	79.8	
> 40 years	66	36.1	21	20.2	
Marital status					0.357^‡^
Single	96	45.5	56	48.3	
Married/stable union	115	54.5	60	51.7	
Race/color					0.064^‡^
White	162	76.8	98	84.5	
Non-white	49	23.2	18	15.5	
Workload					0.180^§^
Up to 40 hours a week	145	68.7	71	61.2	
41 – 80 hours a week	46	21.8	36	31.0	
More than 80 hours a week	20	9.5	9	7.8	
Work relationship					0.180^§^
CLT^||^	129	61.7	83	71.6	
Statutory	58	27.8	23	19.8	
Temporary	18	8.6	6	5.2	
Other	4	1.9	4	3.4	
Position/role					**0.014** ^‡^
Nurse	41	19.4	36	31	
Nursing technician	170	80.6	80	69	
Unit in which they operate					0.297^§^
COVID-19 inpatient unit	24	11.4	11	9.5	
COVID-19 ICU	28	13.3	20	17.2	
COVID-19 emergency	17	8.1	6	5.2	
Other COVID-19 unit	35	16.6	26	22.4	
I work in more than one COVID-19 unit	79	37.4	45	38.8	
I work in a COVID-19 and non-COVID-19 unit	28	13.3	8	6.9	
Work shift					0.448^‡^
Day	77	36.5	46	39.7	
Night	83	39.3	49	42.2	
Mixed	51	24.2	21	18.1	
Job tenure					0.071^‡^
< than 5 years	67	31.8	47	40.5	
5 years and more	144	68.2	69	59.5	

*Results presented in absolute and relative frequencies; ^†^Common mental disorders; ^‡^p-value associated with Fisher’s exact test; ^§^p-value associated with Pearson’s chi-square test; ^||^Consolidation of Labor Laws. Source: research database.

Qualitative data shed light on these results. The association between CMD screening and profiling young adult women showed interfaces with the impacts of COVID-19 on life outside work, in which young people and those with small children were especially weakened by restrictive social distancing measures:

(…) *I don’t socialize with other people* (…) *I’m even afraid to talk, to relate to people* (…) *I really like going out, having fun, parties and that’s not the case anymore. It was generating stress, a feeling of being trapped* (…) (W4).

(…) [before the pandemic] *I came home, lay down and slept. Today, I arrive and my little one is awake. I joke that I have the responsibility to make her sleep* (…) *my mother-in-law came to take care of her, but because I was in two hospitals… fear of contaminating her* (…) (W2).

Nurses, on the other hand, were especially affected by psychological overload related to the responsibilities of care management and the work process in COVID-19 units. Especially in times of major crisis, nurses felt the responsibility to provide minimum conditions of care in a context of adversity:

(…) *a volume of information caused me a lot of anxiety* (…) *even though I wasn’t working, these WhatsApp groups were always updating, questions of scale, of patients who died. My phone kept ringing all the time and everything revolved around COVID-19* (…) *I brought a lot of work home and it [worsened the] anxiety condition* (…) *I kept thinking, “how will it be? No people, no equipment”* (…) (W34).

### Common Mental Disorders and Interface with Nursing Workers’ Health Conditions and Life Habits in Covid-19 Units

From the point of view of workers’ health conditions and lifestyle habits, statistically significant results were evidenced from the association between CMD screening and variables: having a disease; using drugs that they did not use before the pandemic; making use of psychoactive drugs; using more than one class of drugs, including psychoactive drugs and sleep aquality nd regular or very poor eating. These results are presented in Table 2.

**Table 2.** Common mental disorders and the association with nursing workers’ health conditions and life habits in COVID-19 units – Rio Grande do Sul, Brazil, 2021 (n = 327)*.

Variable	CMD screening^†^				p-value^‡^
	No (n = 211)		Yes (n = 116)		
	N	%	N	%
Presence of any disease					**0.003**
No	173	82	78	67.2	
Yes	38	18.0	38	32.8	
More than one type of associated disease					**0.005**
No	210	99.5	110	94.8	
Yes	1	0.5	6	5.2	
Smoking					0.620
No	191	90.5	103	88.8	
Yes	20	9.5	13	11.2	
Physical exercise					0.050
No	109	51.7	73	62.9	
Yes	102	48.3	43	37.1	
Use of medications they did not use before the pandemic					**< 0.001**
No	186	88.2	60	51.7	
Yes	25	11.8	56	48.3	
Use of analgesics, antipyretics, anti-inflammatory drugs					0.161
No	201	95.3	106	91.4	
Yes	10	4.7	10	8.6	
Use of psychoactive drugs					**< 0.001**
No	203	96.2	93	80.2	
Yes	8	3.8	23	19.8	
Use of more than one drug class, including psychoactives					**0.001**
No	209	99.1	107	92.2	
Yes	2	0.9	9	7.8	
Satisfaction with sleep quality					**< 0.001**
Good or very good	87	41.2	11	9.5	
Regular or poor	124	58.8	105	90.5	
Satisfaction with diet quality					**< 0.001**
Good or very good	91	43.1	11	9.5	
Regular or poor	120	56.9	105	90.5	
Belonging to the COVID-19 risk group					0.448
No	196	92.9	105	90.5	
Yes	15	7.1	11	9.5	
Tested positive for COVID-19					0.397
No	157	74.4	81	69.9	
Yes	54	25.6	35	30.1	

*Results presented in absolute and relative frequencies; ^†^Common mental disorders; ^‡^p-value associated with Pearson’s chi-square test. Source: research database.

When triangulating the qualitative data with these results, we identified relationships between mental illness and the presence of one or more diseases, which can be explained by the perception of risk, even among young workers:

(…) *it was a place I didn’t want to work at all, out of fear. We saw many colleagues from other regions dying* (…) (W7).

(…) *I’ve seen several [severe] cases of people who have comorbidities, young people, go to the ICU, with a tube. Many die, I get scared, I have two daughters, they need me a lot* (…) (W12).

Dissatisfaction with sleep and diet quality and the use of medications (especially psychoactive drugs) were elements densely present in participants’ narratives and related to mental health:

(…) *I’ve been eating too much* (…) *and this time change, I can’t adapt. My sleep is very disturbed. I sleep little. I’ve been sleeping four hours, five hours. Today, I slept three hours, because I had to go in at seven o’clock* [in the COVID-19 unit]*, so I get anxious thinking I’m going to lose time* (…) (W1).

(…) *sometimes, I work all night in the hustle, in the tension, I get home and I can’t sleep, I can’t rest, I can’t disconnect from work* (…) *my mind is always working, I had to take medication* (…) (W34).

(…) *insomnia, I leave here, I get home and I can’t sleep. The other day, that feeling of sleep. The food issue. Here,* [COVID-19 unit] *we eat almost nothing. There is no way to eat a lot, water, I drink very little.* (…) *I had to go back to taking antidepressants* (…) (W4).

Nursing workers stressed that the impacts that the pandemic had on their lives and the lack of psychological support also stood out among the stressors (at times, overcoming the fear of contamination):

(…) *I had anxiety attacks that triggered a strong migraine attack. I was away for a week* (…) *it wasn’t the coronavirus, but it was what it made of changes in my life that caused it* (…) (W1).

(…) *the problem is not contamination* (…) *today, what is harming professionals is not the issue of protective equipment, it is the psychological issue. Is the lack of psychological assistance* (…) (W8).

## DISCUSSION

Females prevailed, with a mean age of 35.3 (±9.3) years, white, married or in stable union. There was a predominance of nursing technicians, with 5 years or more of work and a workload of up to 40 hours per week. This profile is in line with findings from other studies conducted in Brazil^(3,4,8)^ and in the international context^(2,12,18,19)^.

CMD was screened in 35.5% of the sample using the SRQ-20. This finding was close to the results of other studies conducted with hospital nursing workers, who obtained CMT screening of 36.7%^(3)^ and 32.6%^(6)^. However, it is higher than that found in research conducted with nursing workers in the hospital context^(4)^ in the international scenario, which included frontline professionals facing the pandemic^(18,19)^. In these studies, CMD screening ranged from 16.2% to 26.09%. This indicates that this prevalence in the sample studied is significant when compared to findings from other studies.

Parallel to these findings, the statements indicate that daily life in covid-19 units provides experiences that may be related to CMD screening. Health workers who assist patients with severe COVID-19 are exposed to stressful elements. They require a routine of physical and psychologically intense care, including the use of PPE. They are exposed to the risk of being contaminated by SARS-CoV-2 and of bringing contamination to their families,^(20)^, which culminates in psychological damage to these workers^(21)^. This context may explain the increased prevalence of CMD in the context of COVID-19 units.

In order to deepen the understanding of the relationships between mental illness and aspects of the sample studied, the first analytical axis showed the interface between CMD screening and participant socio-occupational profile. Statistical associations were evidenced between this variable and females, in the age group up to 40 years of age, which is in the same line with a similar study^(6)^. Qualitative data contribute to the understanding that the pandemic impacted the lives of young individuals responsible for caring for young children, due to distancing/isolation measures, which weakened social life and family support networks. This interface helps to understand the association between CMD screening and variables sex (since, historically, women have focused more directly on this care) and age (since the younger population tends to have small children, dependent on care).

Isolation and fear of bringing SARS-CoV-2 contamination to the family is a stressful factor for frontline health workers^(11)^. A cross-sectional Chinese study conducted with nursing workers from COVID-19 units showed the association between CMD screening and variable having children. Among the stressors that influenced mental illness, the distancing of their families and the fear of containating them^(18)^ stood out.

A statistical association was evidenced between CMD screening and the professional category of nurses. This result is based on other studies that showed an association with the variable being a nursing technician or assistant^(4,6)^. Qualitative data contribute to unveil the meanings of these findings, as they show that responsibilities around care management and teams operated as an important stressor in COVID-19 units.

In the nursing team, some activities are nurses’ prerogative, such as care management. Even if, sometimes, they have less contact with patients, when compared to nursing technicians, nurses are exposed to triggering factors of psychological distress, such as permanent alertness, fear and tension^(4)^. Therefore, it can be considered that the advent of COVID-19 required the organization of a new reality of routines, protocols, bed management, differentiated standard precautionary measures in the context of care management for a patient profile affected by a previously unknown disease. It is possible that this new reality has given an extra burden on nurses.

The second analytical axis showed the interfaces between CMD screening and nursing workers’ health conditions and life habits in COVID-19 units. First, an association was found with variables having some disease and having more than one associated disease, a finding similar to that of another research, carried out with nursing workers, which showed an association between CMD screening and having four or more diagnosed diseases^(4)^. Moreover, a longitudinal study with workers from different professions, who were removed from work due to CMD, showed that 44% of them had multimorbidity (i.e., the presence of more than one associated disease)^(2)^, which strengthens this idea.

This interface between CMD and comorbidities is complemented by qualitative data, because the statements unseede the perception of risk of developing the severe form of COVID-19. It is known that health professionals who work on the front line of coping with the pandemic are concerned about the risks to their health^(11)^.

On the interface between risk perception and mental illness, we mention a longitudinal North American study conducted with health professionals measured their perception of risks at different times throughout the progress of the pandemic, associating it with stress levels. The research showed that the increased perception of risks of contamination by SARS-CoV-2 during the pandemic was associated with an increase in stress levels in the sample studied. In this context, nursing professionals were especially vulnerable and were four times more likely to consider quitting their jobs due to COVID-19^(20)^.

Furthermore, associations were highlighted between CMD screening and variables using medications that did not use before the pandemic, using psychoactive medications, using more than one class of medications, including psychoactive drugs, satisfaction with sleep and diet quality (regular or poor). Qualitative data, in turn, showed that there is a synergy between these elements. Therefore, it is possible that the worsening in nursing workers’ mental health sometimes translates into psychoactive medication, worsening sleep and diet quality.

An integrative literature review study showed that the use of psychoactive substances by nursing workers is a reality present in many scenarios. Predisposing factors for the use of these substances include, in addition to the presence of mental disorders, elements such as the intense workload, precarious working conditions and experiences of suffering, for which the use of these drugs offers psycho-emotional relief. The study also showed that moments of professional or personal crisis demarcated periods in which the use of psychoactive drugs becomes a necessity for these workers^(22)^, which is in line with the results of this study.

The association between CMD screening and worsening sleep quality, or presence of sleep disorders, is highlighted in studies conducted with hospital nursing workers^(3,4)^ and with health professionals from COVID-19 units^(23)^. An integrative review study showed that damage to sleep quality can be a consequence, especially for nursing workers working on the front line of coping with the COVID-19 pandemic^(10)^.

Dissatisfaction with diet quality, evidenced in qualitative and quantitative data, is in line with findings in a cross-sectional study that analyzed the eating habits of hospital nursing workers. The results pointed to reduced consumption of some important food groups and high consumption of soft drinks, artificial juices and sweets^(24)^. Dietary alterations in nursing may be related to everyday aspects of the profession itself, such as shift work, night shifts and working conditions^(25)^.

However, it is important to consider COVID-19 as a cross- country aspect of this phenomenon. There is evidence that the work changes caused by COVID-19 impacted diet quality for frontline workers, being associated with sleep disorders and high levels of psychological symptoms^(26)^. Therefore, this strengthens the idea that this factor may be the result of important changes in the routine of nursing workers when they enter the COVID-19 units, intersecting with their mental health.

Finally, the qualitative findings clarified that, in study participants’ perception, psychological overload and lack of psycho-emotional support operated as some of the main factors involved in their mental illness. It is known that nursing workers’ mental health is especially fragile in the context of the COVID-19 pandemic. The health crisis caused by SARS-CoV-2 has triggered physical, emotional and psychological consequences in these workers^(10)^. This population faces an increased risk of experiencing stress, anxiety, depression, burnout, addiction and posttraumatic stress disorder, which may have long-term psychological implications^(11)^.

The mental health of nursing workers who are at the forefront of coping with COVID-19 requires specialized attention. Health institution managers should enable psychoemotional support to these workers in order to strengthen their resilience^(18)^. The creation of therapeutic listening spaces will be an important measure to be carried out in the post-pandemic period, in which psychological damage among some nursing professionals will perhaps remain as a legacy of their work on the front line.

This study presented as a limitation the fact that data collection occurred during different phases of the pandemic in the state of Rio Grande do Sul. It is believed that participants’ perception was conditioned by the moment they were in with regard to the waves of contamination in the different macro- regions, which assumed unique characteristics. Therefore, it should be considered that different groups of participants responded to the survey at different times of health crisis, which gives the data a possible temporality bias.

## CONCLUSION

The results suggest the presence of mental illness in nursing workers from COVID-19 units, possibly associated with the repercussions of the pandemic on their work process and in their personal lives. These findings contribute to the advancement of nursing in the understanding of how the profession was affected by the health crisis of COVID-19. These results provide support for management actions in hospital health services towards psychoemotional care to these workers, which should also be perpetrated in the post-pandemic period.
